# Multi-Omics Approaches Revealed the Associations of Host Metabolism and Gut Microbiome With Phylogeny and Environmental Adaptation in Mountain Dragons

**DOI:** 10.3389/fmicb.2022.913700

**Published:** 2022-06-28

**Authors:** Wei Zhu, Yin Qi, Xiaoyi Wang, Xiudong Shi, Liming Chang, Jiongyu Liu, Lifeng Zhu, Jianping Jiang

**Affiliations:** ^1^CAS Key Laboratory of Mountain Ecological Restoration and Bioresource Utilization and Ecological Restoration Biodiversity Conservation Key Laboratory of Sichuan Province, Chengdu Institute of Biology, Chengdu, China; ^2^College of Life Sciences, Nanjing Normal University, Nanjing, China; ^3^College of Life Sciences, University of Chinese Academy of Sciences, Beijing, China; ^4^Mangkang Ecological Station, Tibet Ecological Safety Monitor Network, Chengdu, China

**Keywords:** heterogeneous environment, lizard, local adaptation, multi-omics, organ heterogeneity

## Abstract

The molecular basis enabling the adaptation of animals to spatially heterogeneous environments is a critical clue for understanding the variation, formation, and maintenance of biodiversity in the context of global climate change. Mountain dragons (Agamidae: *Diploderma*) thrive in the Hengduan Mountain Region, a biodiversity hotspot and a typical spatially heterogeneous environment. Here, we compare the liver and muscle metabolome and gut microbiome of 11 geographical populations from three *Diploderma* species (*D. iadinum*, *D. yulongsense*, and *D. vela*) after 7 days acclimation in the same laboratory conditions. Amino acid metabolism, particularly the products of the glutathione cycle, accounted for major interspecies variations, implying its significance in genetic differentiation among mountain dragons. Notably, the cold-dwelling *D. vela* and *D. yulongense* populations tended to have higher glycerophosphate, glycerol-3-phosphocholine, and kinetin levels in their liver, higher carnosine levels in their muscle, and higher Lachnospiraceae levels in their gut. Phylogeny, net primary productivity (NPP), and the temperature had the highest explanation rate to the variations in muscle metabolome, liver metabolome, and gut microbiome, respectively, suggesting heterogeneity of biological systems in response to climatic variations. Therefore, we suggested that the organ heterogeneity in environmental responsiveness might be substantial for mountain dragons to thrive in complicated environments.

## Introduction

Almost all animals live in spatially heterogeneous environments (Keller and Seehausen, 2012). Environmental heterogeneity can drive adaptive divergences between populations at both genetic and physiological levels (Rainey and Travisano, 1998; Valladares et al., 2014). These interpopulation variations play a fundamental role in maintaining a species’ genetic or functional diversity and response to climate change (Charmantier et al., 2008; Zhu et al., 2021c). Moreover, adaptive genetic divergence may result in ecological speciation if it causes some form of reproductive isolation (Schluter, 2000; Rundle and Nosil, 2005). Thus, the physiological strategies and underlying molecular basis of environmental adaptation are critical clues for understanding the formation, maintenance, and variation trends of biodiversity in the context of global climate change.

Reptiles have been suggested to be particularly sensitive to climate change due to their poor dispersal capacity (Urban et al., 2013b). These animals have already experienced extensive declines and extinctions worldwide (Sinervo et al., 2010), and climate change has contributed to these threats (Stuart Simon et al., 2004). Correlative climate envelope models even predict that climate change will cause the extinction of 11–49% of endemic reptiles (Thomas et al., 2004), and 20% of lizard species are expected to extinct by 2080 (Sinervo et al., 2010). However, phenotypic plasticity and genetic adaptation are expected to mitigate some of the negative biotic consequences of climate change (Holt, 1990; Urban et al., 2013a). Thus, good knowledge of an environment’s adaptive mechanisms is important for understanding the influences of climate change on these animals and also a precondition for scientific and accurate conservation measures. In this regard, species adapted to typically heterogeneous environments provide a unique opportunity to study how these animals respond to environmental variations. The Hengduan Mountain Region (HMR), located in the southeastern part of the Tibet Plateau, varies spatially in climatic factors (e.g., temperature and precipitation) (Xu et al., 2018), resulting in many different ecosystems (Myers et al., 2000; Lei et al., 2015). It harbors high reptile diversity, including more than ten *Diploderma* (Squamata: Sauria: Agamidae; mountain dragons) species, which are micro-endemic to the dry and hot valleys of HMR (Wang et al., 2020). Currently, the formation of *Diploderma* diversity in HMR has not been studied systematically, and vicariant isolation, ecological divergence, and the low migration capacity of lizards (within 10 km; Southwood and Avens, 2010) are all potential drivers. The distribution areas of the *Diploderma* species are narrow but highly heterogeneous in terms of spatial climatic factors (Zhu et al., 2021c). This makes the *Diploderma* species an ideal model for investigating environmental adaptation at a micro-geographic scale.

Comparative genetics and genomics are major approaches in the study of the mechanisms of environmental adaptation (Savolainen et al., 2013; Kubota et al., 2015). However, the accumulation of stochastic genetic changes in the genome constitutes an obstacle to the screening of environment-related variations, especially for interspecies studies. Additionally, genetic variations may not always explain adaptation processes intuitively due to the limited understanding of their cellular functions (Chang et al., 2020), especially when adaptive traits are determined by multiple genetic loci or mutations are located in non-coding regions. Moreover, not all environmental adaptive traits are caused by changes in DNA sequences; for example, epigenetics, in response to external or environmental factors, can also shape cellular and physiological phenotypic traits by changing cellular gene expression patterns (Bird, 2007; Vogt, 2017). Despite the variability of genetic materials and the complexity of genetic determination, the expression of genetic information in phenotypic traits always relies on the qualitative and quantitative variations in a set of cellular chemical molecules—metabolites, which are the effectors of cellular regulation networks and the molecular basis of phenotypes (Nicholson and Lindon, 2008; Johnson et al., 2016). The whole set of metabolites in a cell, tissue, or organisms is called metabolome, which is at the frontline of the interactions between organisms and the environment (Bundy et al., 2009). Unlike the sequence mutations of genes, the chemical structures of primary metabolites are not easily varied with phylogeny, and species with close phylogenetic relationships likely share the same set of primary metabolites. This facilitates convenient comparisons on environment-related biological constructions and physiological functions between species or populations in the absence of whole-genome data. For example, comparative metabolomics revealed the critical role of metabolic switch in substrates for the thermal adaptation of a Plateau dwelling insect (Zhu et al., 2016; Zhu et al., 2019a). And combined comparative transcriptomic and metabolomic analysis illuminated the involvement of fatty acid metabolism in the highland adaptation across altitudinal songbirds (Xiong et al., 2021). Accordingly, comparative metabolomics provides an alternative approach for studying the adaptation of animals to spatially heterogeneous environments (Shi et al., 2015).

Additionally, the gut microbiome is considered to be the second genome of animals (Zhu et al., 2010). The host genome is highly conserved, and genetic changes within it occur slowly, whereas the gene pool of microbiota is dynamic and can change rapidly in response to the environment by increasing or reducing the abundance of particular microbes, by acquisition of novel microbes, by horizontal gene transfer, and by mutation (Rosenberg and Zilber-Rosenberg, 2018). Increasing numbers of studies have evidenced the contribution of the commensal microbiome to host environmental adaptation (Chevalier et al., 2015; Zhang et al., 2016); a disturbance in the gut microbiome can also lead to animal maladaptation to climate change (Greenspan et al., 2020). For example, gut microbiota promote cold adaptation of Brandt’s voles by increasing host thermogenesis through the activation of cAMP–PKA–pCREB signaling (Bo et al., 2019). Seasonal shifts in gut microbiota composition is in favor of utilization of nitrogen and energy in yaks at harsh cold winter, implying essential role of symbiotic microbiota in high altitude adaptation of animals (Guo et al., 2021). Thus, variations in the microbiome are important aspects that should be considered in the environmental adaptation of animals.

**FIGURE 1:**
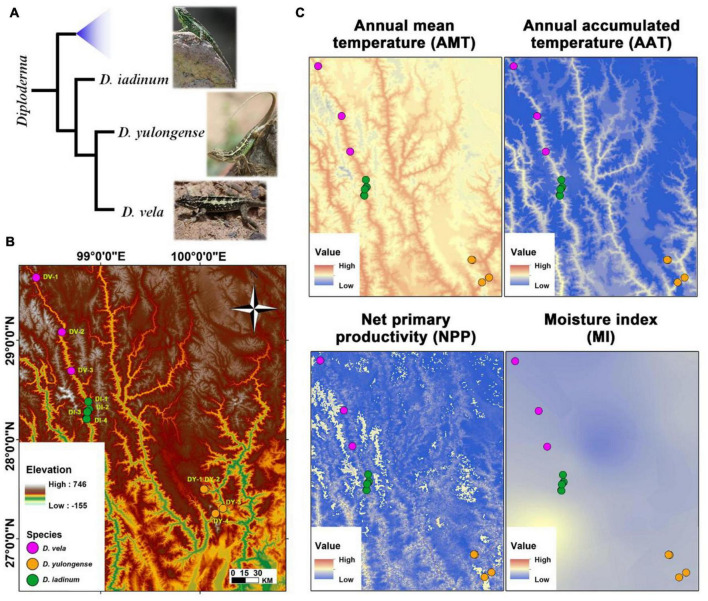
Animal phylogeny, study area, and significant climatic factors. **(A)** The phylogenic relationships of *Diploderma* species (*D. iadinum*, *D. yulongense*, and *D. vela*) (Wang et al., 2020). **(B)** Study area and sample collection sites. **(C)** Maps illustrating the major climatic factors. The white area in the map of net primary productivity (NPP) denotes that data are unavailable.

In this study, environment-related variations in organ metabolism (i.e., liver and hindlimb muscle) and gut microbial community composition were studied in three *Diploderma* species (*D. iadinum*, *D. yulongense*, and *D. vela*). *D. yulongense* and *D. vela* are phylogenetically closer to each other than to *D. iadinum* (Figure 1A; Wang et al., 2020), while *D. iadinum* and *D. vela* are spatially closer to each other (the Lancang River Valley) than to *D. yulongense* (the Jinsha River Valley) (Figure 1B). The associations between biological traits and climatic factors (e.g., temperature and precipitation) were analyzed at a population level (across species and within species). We raise three scientific questions regarding the contributions of phylogeny and climatic factors to animal metabolic and microbiome variations: (1) What factor is more significant, and whether or not there is any organ heterogeneity? (2) do the metabolome and microbiome vary with climatic factors convergently between species? and (3) which are the biological functions of the environment-related variations and their implications in environmental adaptation?

## Materials and Methods

### Habitats and Animals

The *D. vela* (*n* = 15) and *D. iadinum* individuals (*n* = 14) and *D. yulongense* individuals (*n* = 17) were sampled from the dry and hot valley of the Langcang River and the Jinsha River, respectively, in July 2020 (Figure 1B and Supplementary Table 1). The sample information is detailed in Supplementary Table 1. As the study involved invasive experiments, only males were collected. Considering that the animal metabolome and gut microbiome are likely variable to their instant physiological status (e.g., feeding status) and environmental conditions (e.g., the real-time temperature and moisture at the time of collection), the gut content and tissues were not sampled immediately after collected. Instead, all the individuals were acclimated to the same laboratorial condition (24 ± 1°C, L: D = 12: 12) for 7 days before sample collection to diminish the random variations. Our goal was to reveal the inter-populational divergences in animal metabolome and gut microbiome that were potentially associated with phylogeny and climate. Such an acclimation procedure could ensure the authenticity of the differences between geographical populations, despite some information might be lost during this process. During acclimation, each individual was placed in a 29 × 18 × 10 cm plastic container and fed with mealworm (*Tenebrio molitor* larvae) and tap water daily. The feeding behavior was confirmed by daily observation. Following euthanasia with ether, the liver, hind limb muscle, and gut mucosa (from the intestine to the rectum, pooled) were collected and stored at –80°C. Animal procedures were approved by the Animal Care and Use Committee of the Chengdu Institute of Biology, Chinese Academy of Sciences (permit number: 2020-AR-JJP-01).

Environmental factors (i.e., annual mean temperature/AMT, annual precipitation/AP, annual accumulated temperature (> 10°C)/AAT, moisture index/MI, and net primary productivity/NPP) were extracted from Resource and Environment Science and Data Center.^1^ This information is detailed in Supplementary Table 1. The gut microbiome and tissue metabolomics data of *D. vela* have been published previously (Zhu et al., 2021c).

### Metabolic Profiling

After grinding in liquid nitrogen, 50 mg tissue powder was transferred into 1.5 ml Eppendorf tubes with 800 μL precooled methanol: acetonitrile = 1:1 (v/v), followed by ultrasonication for 30 min × 2 and incubation at –20°C for 1 h. After centrifugation at 16,000 g for 20 min (4°C), supernates were transferred into new tubes and freeze-dried. Samples were dissolved in 100 μL acetonitrile: water (1: 1, v/v). After centrifugation at 14,000 g for 15 min (4°C), the supernates were ready for analysis. Extracted supernatants were analyzed by LC (1,290 Infinity LC, Agilent) coupled with quadrupole-time-of-flight mass spectrometry (Triple TOF 5,600 +, AB SCIEX). The details in the metabolic profiling followed the methods described by Zhu et al. (2021c). Metabolite data were processed using XCMS software^2^ and Microsoft Excel (Microsoft, Redmond, WA, United States). Data of impurity peaks from column bleeds were excluded. Metabolites were identified by a combination of molecular weight comparison (molecular ion peak) and MS/MS spectrum comparison to a standard library. The relative abundances/concentrations of metabolites were presented as the ion intensities of their molecular ion peaks.

### 16S rRNA Gene-Based (Full Length) Microbiome Analyses

The PowerSoil^®^ DNA Isolation kit (MO BIO Laboratories, Solana Beach, CA, United States) was used to extract DNA from the samples at room temperature according to the manufacturer’s protocol. A DNA extraction (blank) control was included during DNA isolation. The integrity of the nucleic acids was determined visually by electrophoresis on a 1.0% agarose gel containing ethidium bromide. The concentration and purity of each DNA extraction was determined using a Qubit dsDNA HS Assay Kit (Life Technologies, Carlsbad, CA, United States). The whole region of the 16S rRNA gene was amplified with 27F (5 = –AGRGTTTGATYNTGGCTCAG-3 =) and 1492R (5 = –TASGGHTACCTTGTTASGACTT-3 =) primers, following the method described by Zhu et al. (2021c). A DNA extraction (blank) control was also included during PCR reaction. We used the following PCR thermocycling conditions: 95°C for 5 min, 30 cycles of 95°C for 30 s, 50°C for 30 s, and 72°C for 60 s, with a final extension step at 72°C for 7 min. The products were purified with MagicPure Size Slection DNA Beads (TransGen Biotech, Beijing, China). High-throughput sequencing was performed using the PacBio platform. Sequencing (including the blank control) was performed by Biomarker Technologies Corporation (Beijing, China). The optimized circular consensus sequences (CCS) were obtained after filtering with the threshold of minPasses ≥ 5, minPredictedAccuracy ≥ 0.9, and length between 1,200 and 1,650 bp (lima v1.7.0 and cutadapt 1.9.1). Amplicon sequence variants (ASVs) were obtained after denoising with dada2 (Callahan et al., 2016). Annotation was conducted by querying against SILVA 132 (Quast et al., 2013), and the taxon summary was shown with QIIME2 2020.6 pipeline (Bolyen et al., 2019). The alpha-diversity (e.g., ACE and Shannon index) was calculated in QIIME 2. The dissimilarity matrices (e.g., unweighted and weighted UniFrac distances) were produced by QIIME2 pipeline.

### Statistical Analyses

The influences of phylogeny (presented as genetic distances; Wang et al., 2020) and climatic factors (AMT, ATT, AP, NPP, and MI) on organ metabolome and gut microbiome were analyzed by PERMANOVA (adonis function in the Vegan package) based on R platform (Dixon, 2003). These analyses set metabolites or bacterial taxa abundances as dependent variables, and genetic distances and climatic factors were selected as independent factors. In detail, the beta-diversity of organ metabolomes was presented as Bray-Curtis distance, and the beta-diversity of gut microbiome was presented as Binary Jaccard, Bray-Curtis, Unweighted UniFrac, and Weighted UniFrac distances. The climatic factors and phylogeny (shown as the mean genetic distances to other species) were independent factors. Type I sum of square was used, as our main target was to screen the factors that had the most significant explanation rate to the variations. For each analysis, e.g., influences of phylogeny and climatic factors on beta-diversity of gut microbiota based on Bray-Curtis distances, the model was built by adding independent factors one by one. The sequences of independent factors were determined by ensuring that the factor added at each round could offer the most significant improvement on the total explanation of the total variations of dependent factors. The significance of each independent factors to the model was checked at threshold of *p* < 0.05. This method could avoid the interference of variable autocorrelation on models and reflect the importance order of factors.

Metabolites and bacterial taxa varied with phylogeny or climatic factors were screened. The metabolites or bacteria associated with phylogeny met the threshold of significant difference between any two species (at *p* < 0.05, Mann–Whitney *U*-test). Pearson correlations and Spearman correlations was used simultaneously to screen the metabolites and gut bacteria associated with climatic factors. Valid pairwise correlations met the threshold of *q* < 0.05 or *q* < 0.01 (Pearson correlation and BH correction) and *p* < 0.05 (Spearman correlation). The potential bacterial functions and phenotypes were predicted with PICRUSt2 (Douglas et al., 2020) and BugBase (Ward et al., 2017). However, PiCRUST was developed for human microbiome function and should certainly be used with caution for host groups outside the Mammalia. ANCOVA was conducted to analyze the variations in gut bacterial alpha-diversity and bacterial functions. The intraspecies differences in organ metabolome and gut microbiome between populations were analyzed by PERMANOVA (adonis function in the Vegan package). Principal coordinate analyses (PCoA, based on dissimilarity matrices) were used to visualize the dissimilarity of beta-diversity. Metabolite enrichment analyses were conducted using MetaboAnalyst 5.0.^3^ The Spearman correlation coefficients were calculated for each metabolite–bacteria pair. To ensure the reliability of the results, the coefficients were calculated across all the samples from different species, as well as limited for the samples from the same species. Valid metabolite–bacteria correlations should meet *p* < 0.001 across samples and *p* < 0.01 at least in two species. Correlation networks were constructed on Cytoscape 3.5.0. Other graphs were drawn using Graphpad prism 5 and ggplot2, an R package (Wickham, 2009).

## Results

### Environmental Heterogeneity of the Diploderma Distribution Range

The climatic factors (i.e., AMT, AAT, NPP, AP, and MI) vary spatially across the distribution range of *Diploderma* species (Figure 1C and Supplementary Table 1). The AMT and AAT share similar variation pattern, which is different from that of NPP and AP (Supplementary Figure 1). The collection sites for *D. vela* tended to have lower temperature, MI, and NPP than that of the other two species, and the intraspecies variations in temperature is prominent for *D. vela* and *D. yulongense*.

### Variations in Organ Metabolome Across Species

**FIGURE 2:**
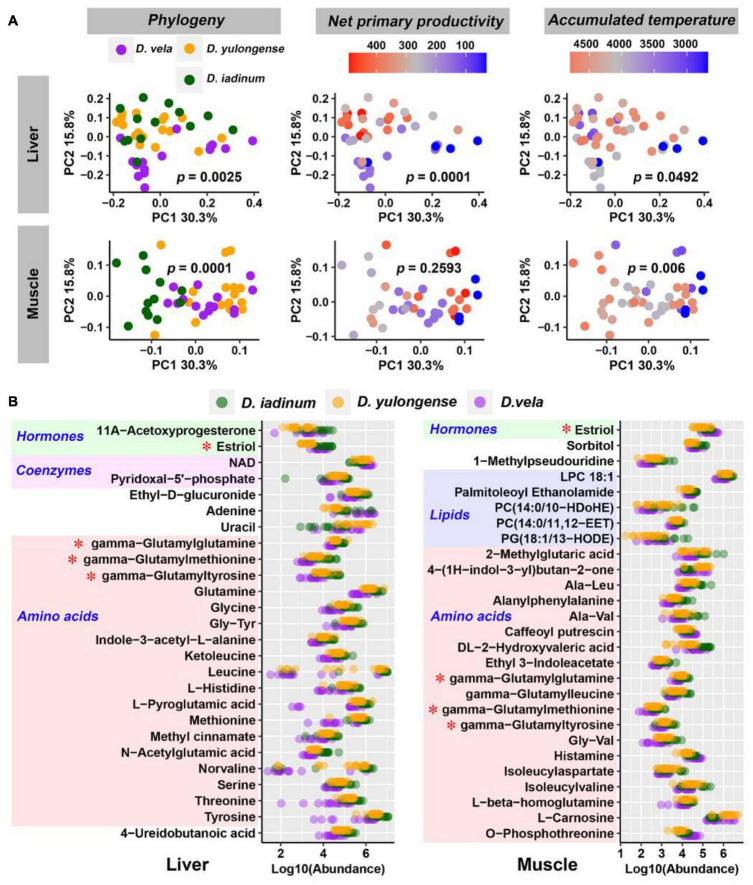
Influences of host phylogeny and climatic factors on the liver and muscle metabolic profiles. **(A)** PCoA scatter plots presenting the significance of phylogeny and climatic factors on variations in organ metabolome. The *p*-values of PERMANOVA were labeled. **(B)** Metabolites varied in abundance between any two *Diploderma* species (*p* < 0.05, Mann–Whitney *U*-test). Red asterisks denote the metabolites shared by the liver and muscle.

*D. iadinum*, *D. yulongense*, and *D. vela* differed significantly in their liver and hindlimb metabolomes (Supplementary Table 2). However, for the liver metabolome, *D. yulongense* was more similar to *D. iadinum* than to *D. vela* (Supplementary Figure 2), despite *D. yulongense* and *D. vela* being more adjacent in phylogeny. In fact, while phylogeny contributed significantly to variations in liver metabolome (*R*^2^ = 0.072, *p* = 0.0025), NPP explained the variations better than phylogeny (*R*^2^ = 0.126, *p* = 0.0001) (PERMANOVA; Table 1 and Figure 2A). The similarity between species was consistent with their genetic distances (Supplementary Figure 2). Additionally, phylogeny had the highest explanation rate (*R*^2^ = 0.175, *p* = 0.0001) regarding the variations in muscle metabolome (PERMANOVA, Table 1 and Figure 2A). The other significant contributors included AAT and AP.

**TABLE 1:** The permutation ANOVAs on liver and muscle metabolomes (Bray-Curtis distance, permutations = 9,999) for the best models.

Tissue	Factors	F model	*R* ^2^	*p*
Liver	NPP	6.0752	0.1263	0.0001
	Phylogeny	3.4673	0.0721	0.0025
	AAT	1.9947	0.0415	0.0492
	AP	2.1221	0.0441	0.0371
Muscle	Phylogeny	8.8817	0.1748	0.0001
	AAT	2.6577	0.0523	0.0060
	AP	3.1607	0.0622	0.0027
	AMT	1.8952	0.0373	0.0438

Most phylogeny-associated metabolites did not overlap with those associated with climatic factors (Supplementary Figure 3A). In both liver and muscle, phylogeny-associated metabolites were primarily amino acids and their derivates, most of which shared similar variation patterns across species (metabolite abundance: *D. iadinum* > *D. yulongense* > *D. vela*) (Figure 2B). Functional enrichment analyses against the KEGG database highlighted the potential involvement of hepatic purine metabolism, glycine and serine metabolism, ammonia recycling, and methionine metabolism in genetic differentiation of *Diploderma* species (Supplementary Figure 3B). Notably, three gamma-glutamyl dipeptides (i.e., gamma-glutamylglutamine, gamma-glutamylmethionine, and gamma-glutamyltyrosine) were shared by the liver and muscle.

**FIGURE 3:**
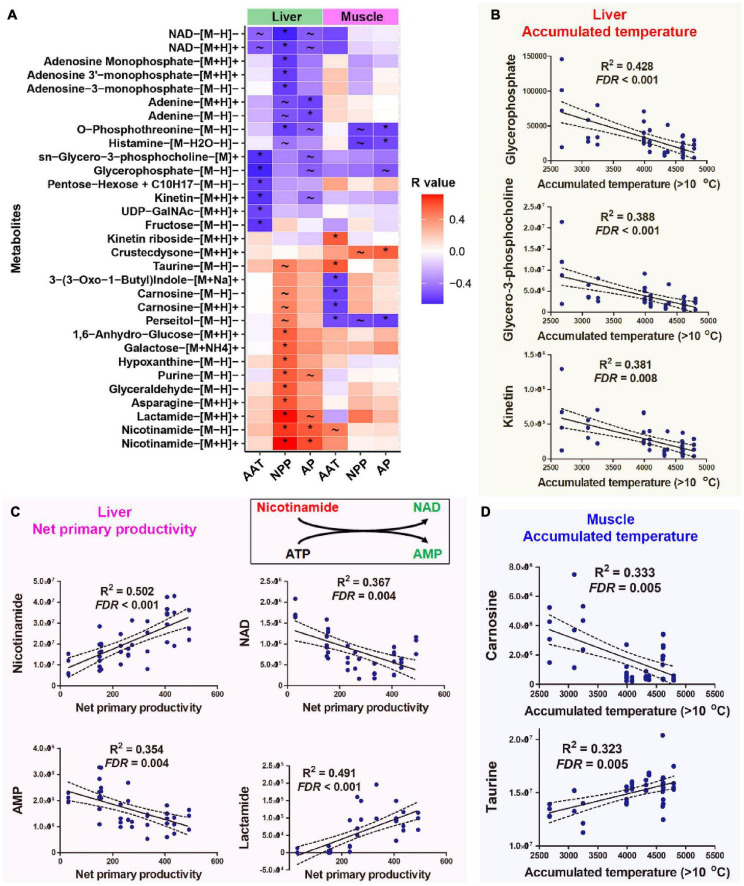
Host metabolites associated with climatic factors. **(A)** Heatmap presenting the correlations (*R*-value) between host metabolites and climatic factors. Climatic factors were limited to those contributing significantly to the variations in organ metabolome (*p* < 0.05, PERMANOVA). The metabolites presented were significantly correlated with (*q* < 0.01, Pearson correlation; *p* < 0.05, Spearman correlation) at least one climatic factor. *q < 0.01 for Pearson correlation and *p* < 0.05 for Spearman correlation; ∼*q* < 0.05 for Pearson correlation and *p* < 0.05 for Spearman correlation. **(B)** Correlations between AAT and liver metabolites. **(C)** Correlations between NPP and liver metabolites. **(D)** Correlations between AAT and muscle metabolites. The abundances of metabolites were presented as peak area, which had no unit.

NPP and AAT were the primary climatic factors associated with the variation in the liver metabolome. Hepatic metabolites showing strong correlations (*q* < 0.01, Pearson correlation; *p* < 0.05, Spearman correlation) with AAT included glycerol-3-phosphocholine, glycerophosphate, kinetin, and fructose (Figure 3A). Their levels were higher in individuals that inhabited cooler environments (Figure 3B). Compared to other climatic factors, there were more metabolites associated with NPP in the liver (Supplementary Figure 3A); this finding is consistent with the high explanation rate of NPP for liver metabolome. These metabolites highlighted metabolism, nicotinamide metabolism, ammonia recycling, urea cycle, and numerous amino acid metabolisms (Supplementary Figure 3B). The abundance of nicotinamide, NAD, and AMP, which participated in the same metabolic reaction (interconversion between nicotinamide and NAD), showed correlations with NPP level (Figure 3A). Lower NPP was associated with higher NAD and lower nicotinamide in the liver and vice versa (Figure 3C).

AAT and AP were the major climatic factors associated with variations in muscle metabolome. Enrichment analysis highlighted the potential association between muscle histamine metabolism oxidation of branched-chain fatty acids and environmental AAT (Supplementary Figure 3B). Notably, muscle carnosine and perseitol showed a strong negative correlation with environmental AAT, while taurine and kinetin riboside positively correlated with AAT (Figure 3D). Muscle metabolites associating with AP included persecutor, O-phosphothreonine, and histamine (Figure 3A). Only a few metabolites correlated with environmental AAT and NPP simultaneously (Supplementary Figure 4).

### Variations in Gut Microbiome Across Species

**FIGURE 4:**
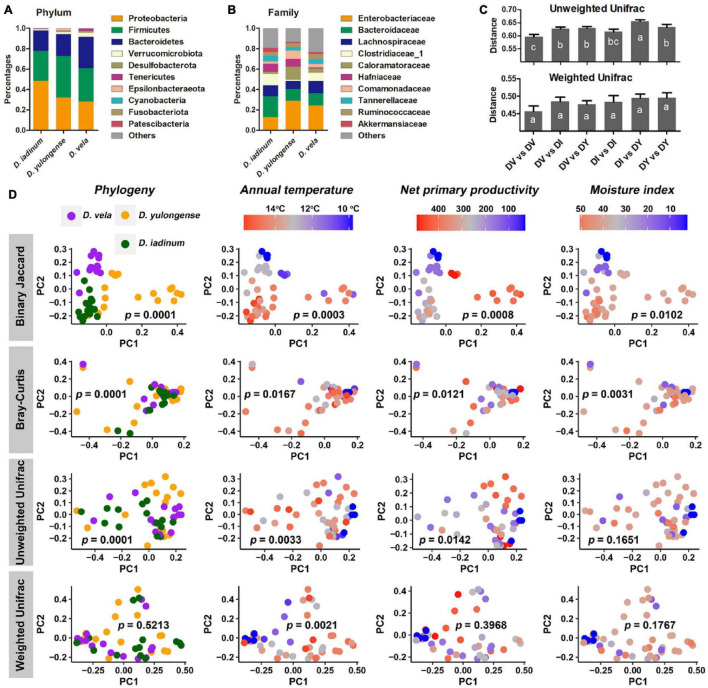
Influences of host phylogeny and climatic factors on the gut bacterial community structure of mountain dragons. **(A,B)** Composition of the microbiome at phylum **(A)** and family **(B)** levels. **(C)** Interspecies distances of the gut microbiome. **(D)** PCoA scatter plots presenting the significance of phylogeny and climatic factors on variations in the gut microbiome (interpopulation analyses across species). The *p*-values of PERMANOVA were labeled.

**FIGURE 5:**
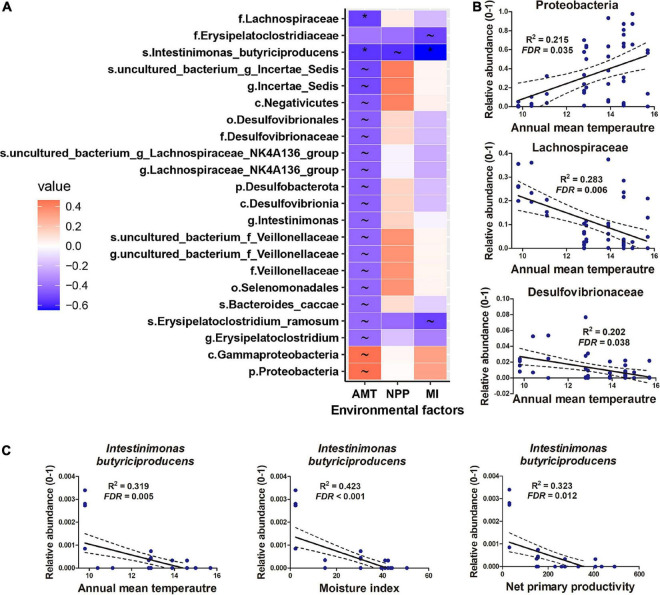
Gut microbiomes associated with climatic factors. **(A)** Heatmap presenting the correlations (*R*-value) between gut microbes and climatic factors. Climatic factors were limited to those contributing significantly to the variations in the gut microbiome (*p* < 0.05, PERMANOVA). *q < 0.01 for Pearson correlation and *p* < 0.05 for Spearman correlation; ∼*q* < 0.05 for Pearson correlation and *p* < 0.05 for Spearman correlation. **(B,C)** Correlations between microbes and environmental factors.

Proteobacteria, Firmicutes, and Bacteroidetes dominated the gut microbiome of *Diploderma* species (Figure 4A), and Enterobacteriaceae, Bacteroidaceae, and Lachnospiraceae were their most abundant bacterial families (Figure 4B). Neither species nor climatic factors had significant influences on the alpha-diversity of the gut microbiota (ANCOVA, Supplementary Table 3). The interspecies similarity of bacterial community structure was not consistent with the genetic distances (unweighted UniFrac distance and weighted UniFrac distance, Figure 4C). When bacterial phylogenetic relations were not considered (Binary Jaccard and Bray-Curtis distances), phylogeny was the primary contributor to the variation in gut microbiota and environmental AMT, NPP, and MI significant contributors (PERMANOVA, Table 2 and Figure 4D). When bacterial phylogenetic relations were considered, phylogeny still had the highest explanation rate (*R*^2^ = 0.122) to the gut microbiota community if ASV abundance was ignored (unweighted UniFrac distance). However, when ASV abundance was taken into consideration (weighted UniFrac distance), AMT was the only significant contributor (*R*^2^ = 0.125) to the variation in the gut microbiota community (Table 2 and Figure 4D). Additionally, interspecies differences no longer existed (Supplementary Table 2). No bacterial groups or ASVs showed significant variations between species (Kruskal–Wallis test at the threshold of *p* < 0.05). Instead, many bacterial groups, including Lachnospiraceae, Desulfovibrionaceae, and Veillonellaceae families, negatively correlated with environmental AMT, while Proteobacteria and Gammaproteobacteria positively correlated with it (Figures 5A,B). Notably, *Intestinimonas butyriciproducens* negatively correlated with environmental AMT, NPP, and MI (*q* < 0.05 in Pearson correlation and *p* < 0.05 in Spearman correlation, Figure 5C). There were no significant associations between any environmental factors and bacterial functions predicted by COG^4^ and KEGG databases.^5^ However, environmental MI was positively correlated with some bacterial phenotypes (i.e., potentially pathogenic, contains mobile elements, and facultative anaerobic) predicted by BugBase (Supplementary Figure 5).

**TABLE 2:** The permutation ANOVAs on symbiotic microbiota (permutations = 9,999) for the best models.

Distance type	Factors	F model	*R* ^2^	*p*
Binary Jaccard	Phylogeny	2.3528	0.10652	0.0001
	AMT	1.5025	0.03401	0.0003
	NPP	1.4711	0.03330	0.0008
	MI	1.2865	0.02912	0.0102
Bray-Curtis	Phylogeny	1.8353	0.08564	0.0001
	MI	1.4406	0.03361	0.0031
	NPP	1.3532	0.03157	0.0121
	AMT	1.3262	0.03094	0.0167
Unweighted UniFrac	Phylogeny	2.8262	0.12229	0.0001
	AMT	2.2056	0.04772	0.0033
	NPP	1.8763	0.04059	0.0142
Weighted UniFrac	AMT	5.5076	0.12475	0.0021

### Intraspecies Variations Associated With Thermal Adaptation

**FIGURE 6:**
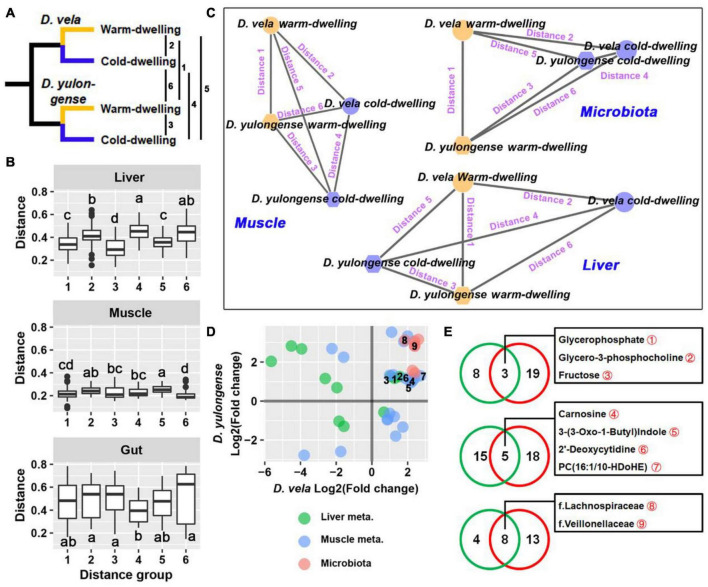
Intraspecies comparisons between cold- and warm-dwelling populations. **(A)** *D. vela* and *D. yulongense* individuals could be divided into cold- (DV-1, DY-1, and DY-2) and warm-dwelling populations (DV-2, DV-3, DY-3, and DY-4) according to the annual temperature. The distances between populations ranged from 1 to 6. **(B)** Distances between cold- and warm-dwelling species. Metabolome: Bray-Curtis distance; microbiome: weighted UniFrac distance. **(C)** Topological networks present the relative distances between groups. Note that a cold environment drives the opposite, parallel, and concurrent variations in the liver, muscle, and gut microbiome, respectively, between *D. vela* and *D. yulongense*. **(D)** Point plot presenting the variation patterns of metabolites and microbes, which differed significantly between cold- and warm-dwelling populations in both species. The horizontal and vertical axes denote the fold changes of cold- to warm-dwelling populations in *D. vela* and *D. yulongense*, respectively. Points with numeric numbers are annotated in panel **(E)**. **(E)** Venn plots presenting the number of metabolites associated with the environmental temperature. Green circle, metabolites or microbes screened by correlation analyses across species (*q* < 0.05 for Pearson correlation and *p* < 0.05 for Spearman correlation); red circle, metabolites or microbes filtered by intraspecies comparisons between cold- and warm-dwelling populations (*p* < 0.05 for Mann–Whitney *U*-test in both species).

For both *D. vela and D. yulongense*, there are populations (DV1, DY1, and DY2) inhabiting environments with more than 2^°^C lower in AMT than other populations from the same species (Figure 6A and Supplementary Figure 6). These populations were classified to be cold-dwelling populations (AMT < 11°C), while the other populations of these two species were classified as warm-dwelling populations (AMT > 12°C). Pairwise distances were calculated between cold-dwelling and warm-dwelling populations of these two species (detailed in Figures 6B,C). For liver metabolome, the distance between two cold-dwelling populations was longer than that between the two warm-dwelling populations (Figure 6B). A topological network suggested that the orientations from warm- to cold-dwelling populations were contradictory between these two species (Figure 6C). The distance between the two cold-dwelling populations was maintained for muscle metabolome compared to that between warm-dwelling populations (Figure 6B). The topological network suggested a paralleled variation orientation of cold adaptation between these two species. The weighted UniFrac distance between two cold-dwelling populations was the shortest (Figure 6B). Its topological network indicated a convergence of gut microbiota in cold-dwelling populations (Figure 6C).

The organ metabolites or gut bacteria that differed between cold- and warm-dwelling populations in both species were screened (Figure 6D). For the liver metabolome, most of these metabolites exhibited divergent variation trends between *D. vela* and *D. yulongense* (Figure 6D), except for glycerophosphate, glycerol-3-phosphocholine, and fructose (Figure 6E). For the muscle metabolome, however, more metabolites exhibited consensus variation trends between the two species (Figure 6D), including carnosine and 2′-deoxycytidine (Figure 6E). For gut microbiomes, all screened taxa presented consensus variation trends between *D. vela* and *D. yulongense*. This included the Lachnospiraceae and Veillonellaceae (Figures 6D,E).

### Correlation Networks Analysis

The correlations (*q* < 0.05 for Pearson correlation and *p* < 0.05 for Spearman correlation) between organ metabolites, gut bacterial taxa, and environmental factors were analyzed, and ten sub-networks were constructed (Supplementary Figure 7). Liver core metabolites (correlated with more than four bacterial taxa) included glycerophosphate, propionyl carnitine, kinetin, carnitine, creatinine, glutaric acid, 3-phenyllactic acid, and 4-guanidinobutyric acid. Muscle core metabolites included taurine, cholest-4,6-dien-3-one, kinetin-riboside, and beta-homothreonine. Core bacterial taxa were p.Proteobacteria, c.Gammaproteobacteria, o.Enterobacterales, f.Comamonadaceae, g.Comamonas, and o.Burkholderiales. Notably, metabolites associated with environmental AAT and bacteria associated with AMT were involved in the same correlation network.

## Discussion

### The Primary Factors Shaping the Organ Metabolome and Gut Microbiome

The most significant determinators on muscle, liver, and gut microbiome of *Diploderma* were phylogeny, NPP, and temperature, respectively. The influence of phylogeny on metabolism is predictable, as species with distant phylogenic relationships accumulate more genetic divergences and thus a more differentiated metabolism (Ma and Zeng, 2004). The metabolic pattern in muscle is associated with locomotive endurance, explosive force, and the locomotor mode of animals and these physiological functions play critical roles in speciation (Hedrick et al., 2020). An interesting finding was that the impact of phylogeny on metabolome exhibited organ heterogeneity. This might be explained by their different sets of organ-specific enzymes (e.g., isoenzymes), which might exhibit different evolutionary rates or varied expression plasticity in response to different environments (Pazzagli et al., 1998; Kryuchkova-Mostacci and Robinson-Rechavi, 2015). Many studies highlight that host phylogeny and diet are the two main factors influencing animal gut microbiota (Ley et al., 2008a,b). Consistently, our study also suggested that the gut bacterial taxa differed between *Diploderma* species (unweighted UniFrac distance). However, when taxon abundance was considered, host phylogeny was no longer a determinator. These results suggested that host phylogeny and environmental variations were mainly responsible for the gut microbiome’s taxonomic and abundance variations.

NPP was a significant determinator for the liver and muscle metabolomes and gut microbiome of mountain dragons. The relevance between primary productivity and the overall metabolic rate has been well documented in vertebrates (Lovegrove, 2000; Shi et al., 2015), and evidence from field studies suggests that a large fraction of observed variations in animal metabolism is attributable to variations in primary productivity rather than direct temperature (Tieleman et al., 2003). This is consistent with our observations in *Diploderma*, whose organ metabolome varies with environmental NPP significantly. As all the individuals in our study had been provided with enough food for 7 days, this association was unlikely due to their different nutrition statuses. Alternatively, it implied potential evolutionary adaptation or adaptive plastic response. In the *Diploderma* lizards, the associations between NPP and metabolome were more significant in the liver than in the muscle. This is reasonable, as the liver plays a central role in metabolic regulation to meet the energy requirements of different organs (Han et al., 2016; Zhu et al., 2021b), and acts a major storage space for resources in fish, amphibians, and reptiles (Derickson, 1976; Zhu et al., 2019b). The associations between environmental NPP and the gut microbiome of mountain dragons might be mediated by food availability and diet compositions, both of which could shape the host’s gut microbiome (Ley et al., 2008a,b).

Like NPP, temperature could also influence the liver and muscle metabolome and gut microbiome of mountain dragons. Environmental temperature imposes selective solid pressure on animals. The metabolome is at the forefront of life in coping with thermal stress. For example, the accumulation of cryoprotectants (e.g., glycerol and proline) and antioxidant metabolites (e.g., glutathione) reinforces the tolerance of thermal-acclimated animals to freezing (Koštál et al., 2012). In *Diploderma* lizards, the muscle was more responsive to variations in environmental temperatures than the liver. Cold-dwelling *D. vela* and *D. yulongense* individuals shared more metabolic variations in the muscle than the liver when compared to their respective warm-dwelling counterparts. The gut bacterial community varied with environmental temperature most significantly in mountain dragons. The influence of temperature on the animal commensal microbiome has been supported by many studies (Fan et al., 2013; Zhu et al., 2021a), and fluctuations in symbiotic microbes have been suggested to play a role in host thermal adaptation (Bo et al., 2019; Guo et al., 2021). Interestingly, we observed convergence in the gut microbiome of cold-dwelling populations from two *Diploderma* species. Diet-driven convergence of the gut microbiome is common to animals (Muegge et al., 2011), while evidence for environment-related convergences of the commensal microbiome is relatively scarce (Zhang et al., 2016). Whether temperature influenced the gut microbiome of *Diploderma* directly required further investigations. For example, it is possible that microorganisms adapted to cold conditions are less diverse than those in warm places or because the diet in cold areas is less varied, which determines a more homogeneous microbiota in these conditions.

**FIGURE 7:**
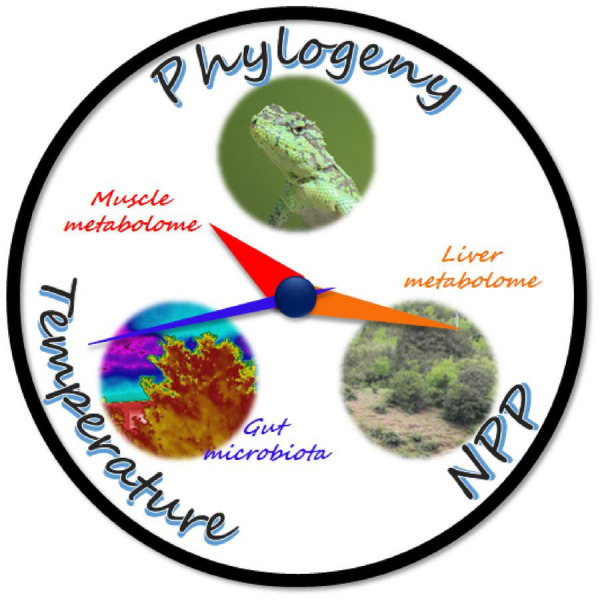
Schematic diagram of the results. The hour, minute, and second hands of the clock symbolize the muscle, liver, and gut microbiomes of the mountain dragons, respectively, and they are pointing to their respective major determinators. The movement speed of the clock hands symbolizes the variability, whether genetic or plastic, of organ metabolome and gut microbiome to the climatic factors. The faster they move, the more variable they are.

Collectively, our results suggested that organ systems might differ in their variability, whether genetic or plastic, to climatic factors. Muscle metabolism predominantly reflects the phylogenetic relationships between species and has the lowest environmental variations. By contrast, liver metabolism exhibits a higher correspondence to environmental factors, especially NPP, but phylogeny is still a significant determinator in the liver metabolome. For the gut microbiome, however, the quantitative traits of the microbiome were only associated with environmental temperature (see a graphic summary in Figure 7).

### Potential Metabolic Adaptation to a Spatially Heterogeneous Environment

Amino acids and dipeptides were highly variable between species, implying that amino acid metabolism underwent remarkable genetic differentiation during the speciation. Notably, three gamma-glutamyl dipeptides (i.e., gamma-glutamylglutamine, gamma-glutamylmethionine, and gamma-glutamyltyrosine) differed between species in both liver and muscle. These metabolites are products of the glutathione cycle, and their levels indicate the oxidative status in tissue (Zierer et al., 2016). Additionally, gamma-glutamylglutamine plays a role in regulating neurobehavioral, e.g., fearfulness (Puurunen et al., 2018). Their variations implied physiological and behavioral divergences during the speciation of mountain dragons. Although their variation patterns between populations were not correlated with current climatic factors, they might be associated with ecological speciation from the perspective of historical climates or presumably involved in topographical adaptations.

The inverse variations in nicotinamide and NAD in the liver were the most significant metabolic variations associated with NPP. Populations with low environmental NPP had higher NAD and lower nicotinamide levels. Nicotinamide is the precursor for NAD biosynthesis (Magni et al., 2004). The cellular NAD level is a critical target for regulating energy expenditure (Cantó et al., 2009). And higher NAD levels are indicative of a metabolic shift from energy storage/anabolism to energy mobilization/catabolism, and vice versa (Revollo et al., 2007). Additionally, NAD is required for the catalytic activity of sirtuins (Imai and Armstrong, 2000; Houtkooper et al., 2010), the deacetylases that promote glucose and lipid metabolism and mediate metabolic transcriptional adaptations linked to nutrition scarcity (Imai and Armstrong, 2000; Canto et al., 2012). Accordingly, the negative correlation between NAD level NPP might be a compensation strategy for the spatial variations in NPP.

The reorganization of phospholipid composition is a common strategy for thermal adaptation in animals (Reynolds et al., 2014). The levels of hepatic glycerol-3-phosphocholine and glycerophosphate, two intermediates in phospholipid metabolism, were negatively correlated with environmental AAT in mountain dragons. These two metabolites were again highlighted in intraspecies comparisons, showing a higher abundance in the cold-dwelling populations. It suggests that phospholipid metabolism might be involved in the adaptation of mountain dragons to spatial variations in temperatures. The hepatic kinetin level was also negatively correlated with environmental AAT. This metabolite was reported to be a cryoprotectant for animal cells with antioxidation function (Zadeh Hashem and Eslami, 2018). Cold-dwelling populations tended to have higher muscle carnosine levels at both interspecies and intraspecies levels. Carnosine is highly concentrated in muscle with biological activities including antioxidation and preventing the formation of advanced glycation end-products (Boldyrev et al., 2013). Notably, carnosine can promote the heat denaturation of glycated protein (Yeargans and Seidler, 2003); thus, we presumed that a higher carnosine level could compensate for the reduced capacity in clearing glycated protein at cold conditions.

### Association of Gut Microbes With Spatially Heterogeneous Environments

Gut Proteobacteria and Gammaproteobacteria abundance was positively correlated with environmental AMT. The variation trends of Proteobacteria vary between animal taxa. In insects, temperature increases have been associated with increased relative abundances of Proteobacteria (Moghadam et al., 2018). In the gut microbiome of the Chinese giant salamander, however, the abundance of Proteobacteria decreased with a rise in temperature (Zhu et al., 2021a). These variations’ biological significance or outcomes to the host have not been illuminated. In contrast to Proteobacteria, the families Lachnospiraceae, Desulfovibrionaceae, and Veillonellaceae tended to be more abundant in cold-dwelling populations of mountain dragons. Members of the Lachnospiraceae family are suggested to be beneficial to the host (Meehan and Beiko, 2014) by promoting short-chain fatty acids, converting primary bile acids to secondary ones, and facilitating colonization resistance against intestinal pathogens (Sorbara et al., 2020). Therefore, its enrichment in the gut of cold-dwelling *Diploderma* populations could be beneficial to the host in adapting to a challenging environment. Our results also suggested that increasing environmental moisture was accompanied by an increase in potential pathogenic and facultative anaerobic bacteria in the gut of mountain dragons (Supplementary Figure 5). Warming and high humidity favor the spread of pathogens in this environment (Bosch et al., 2007). Further studies are required to confirm the association between gut pathogen abundance and environmental moisture in mountain dragons and clarify how this association may influence the existence and distribution of mountain dragons in the HMR. Another noticeable bacterium in mountain dragons was *Intestinimonas butyriciproducens* due to its properties of butyrate production and host metabolic regulation (Kang, 2018). Its abundance negatively correlates with environmental temperature, moisture, and primary productivity. Further functional studies are required to give a mechanistic insight into the role of symbiotic microbiota in host adaptation to environmental variations.

Our results highlighted robust quantitative correlations between host metabolite levels and gut microbe abundances in *Diploderma* lizards, suggesting intimate interactions between host metabolism and gut microbes. Many of these metabolites and microbes were also associated with climatic factors, particularly temperature. It implied coordinated variations in host metabolism and gut microbiota with climatic factors. Thus, our results indicated the significance of the concept of holobiont in investigating the influence of climate on biodiversity.

## Conclusion

Here, we investigated the relationship between variations in organ metabolism and gut microbiota and climatic factors in mountain dragons. The host metabolomes and gut microbiome displayed distinct variability with environmental variations, and their variations were associated with different climatic characteristics. This organ heterogeneity might be important for mountain dragons to thrive in complicated environments. We also observed convergence in the gut microbiome of cold-dwelling populations between species. Our results using the multi-omics approach provided some details regarding the interaction between holobiont and the environment, which might shed some light on the mechanisms underlying evolutionary adaptation in animals.

## Data Availability Statement

The raw data of 16S rRNA gene sequences have been submitted to Genome Sequence Archive (CRA005166) at https://ngdc.cncb.ac.cn/gsub/.

## Author Contributions

WZ, JJ, and LZ conceived the project. YQ and XS collected the samples. WZ, YQ, and XS performed the experiments. WZ, XW, LC, and JL analyzed the data. WZ and LZ wrote the manuscript. All authors approved the final version of the manuscript.

## Conflict of Interest

The authors declare that the research was conducted in the absence of any commercial or financial relationships that could be construed as a potential conflict of interest.

## Publisher’s Note

All claims expressed in this article are solely those of the authors and do not necessarily represent those of their affiliated organizations, or those of the publisher, the editors and the reviewers. Any product that may be evaluated in this article, or claim that may be made by its manufacturer, is not guaranteed or endorsed by the publisher.

## Footnotes

1
http://www.resdc.cn/Default.Aspx

2
https://xcmsonline.scripps.edu

3
https://www.metaboanalyst.ca/faces/ModuleView.xhtml

4
http://www.ncbi.nlm.nih.gov/COG

5
https://www.kegg.jp/kegg/
