# Supplementary material for: Multi-Omics Approaches Revealed the Associations of Host Metabolism and Gut Microbiome With Phylogeny and Environmental Adaptation in Mountain Dragons
Source: Front Microbiol. 2022 Jun 28;13:913700. doi: 10.3389/fmicb.2022.913700 (PMC9273973; doi:10.3389/fmicb.2022.913700)
Supplement: Supplementary file 1 [file Data_Sheet_1.pdf]

Table S1. Sample information.

Species	Population	Longitude	Altitude	NPP ($g/m^2/a$)	AMT (°C)	AP (mm)	MI	AAT (d.°C)	Sample sizes	Sample sizes of experiment		
										Liver metabolome	Muscle metabolome	Gut microbiome
D. iadinum	DI-1	98.876551	28.379953	27.42	13.9	661.3	41.13	4373.4	3	2	2	3
D. iadinum	DI-2	98.877256	28.301292	25.94	15	719.9	43.44	4616.3	3	3	2	3
D. iadinum	DI-3	98.864937	28.274829	23.08	15.7	730.1	44.11	4796.5	4	4	4	4
D. iadinum	DI-4	98.857663	28.209764	33.30	13.9	818.6	50.57	4322.6	4	4	4	4
D. yulongense	DY-1	100.040027	27.500829	49.23	10.4	806.3	39.49	3098.3	3	3	3	3
D. yulongense	DY-2	100.049606	27.498015	43.55	11.1	810.2	39.69	3248.1	3	3	3	3
D. yulongense	DY-3	100.232631	27.305074	40.78	14.6	885.9	42.38	4616.6	6	6	6	6
D. yulongense	DY-4	100.155211	27.253132	--	14.6	867.5	39.9	4607.3	5	5	5	5
D. vela	DV-1	98.350782	29.630491	3.05	9.8	544.9	2.26	2675.6	4	4	4	4
D. vela	DV-2	98.609462	29.081526	15.01	12.8	637.7	15.09	3991	7	7	7	7
D. vela	DV-3	98.704412	28.690494	15.58	12.9	660.2	30.51	4084.6	4	4	4	4

Table S2 Interspecies difference in organ metabolome and gut microbiota (pairwise PERMANOVA, permutations = 99999).

Species	Tissue	Distance type	*p* Value
D. yulongense* vs *D. vela	Liver	Bray-Curtis	< 0.001
	Muscle	Bray-Curtis	< 0.001
	Gut microbiota	Bray-Curtis	<0.001
		Unweighted Unifrac	0.055
		Weighted Unifrac	0.332
D. yulongense* vs *D. iadinum	Liver	Bray-Curtis	0.003
	Muscle	Bray-Curtis	< 0.001
	Gut microbiota	Bray-Curtis	<0.001
		Unweighted Unifrac	0.006
		Weighted Unifrac	0.254
D. vela* vs *D. iadinum	Liver	Bray-Curtis	< 0.001
	Muscle	Bray-Curtis	< 0.001
	Gut microbiota	Bray-Curtis	<0.001
		Unweighted Unifrac	0.019
		Weighted Unifrac	0.108

Table S3 Alpha-diversity of gut microbiota.

Source		Df	F	*p*	Source		Df	F	*p*
**Species**	Features	2	1.94	0.16	**NPP**	Features	1	1.54	0.22
	ACE	2	1.94	0.16		ACE	1	1.54	0.22
	Chao1	2	1.94	0.16		Chao1	1	1.54	0.22
	Simpson	2	1.71	0.20		Simpson	1	2.23	0.15
	Shannon	2	1.89	0.17		Shannon	1	2.34	0.14
	PD_whole_tree	2	2.17	0.13		PD_whole_tree	1	0.62	0.44
**ATM**	Features	1	0.73	0.40	**AP**	Features	1	0.43	0.52
	ACE	1	0.73	0.40		ACE	1	0.43	0.52
	Chao1	1	0.73	0.40		Chao1	1	0.43	0.52
	Simpson	1	1.56	0.22		Simpson	1	0.83	0.37

	Shannon	1	1.71	0.20		Shannon	1	0.98	0.33
	PD_whole_tree	1	0.07	0.80		PD_whole_tree	1	0.01	0.92
**AAT**	Features	1	0.73	0.40	**MI**	Features	1	0.07	0.79
	ACE	1	0.73	0.40		ACE	1	0.07	0.79
	Chao1	1	0.73	0.40		Chao1	1	0.07	0.79
	Simpson	1	1.57	0.22		Simpson	1	0.33	0.57
	Shannon	1	1.70	0.20		Shannon	1	0.38	0.54
	PD_whole_tree	1	0.08	0.78		PD_whole_tree	1	0.13	0.73

Table S4 *D. yulongense* intraspecies difference in organ metabolome and gut microbiota (pairwise PERMANOVA, permutations = 99999). Note that DY1 and DY2 sites were adjacent to each other and they shared similar climatic factors, thus they were treated as one population.

**Population**	**Tissue**	**Distance type**	***p* Value**
**DY1&2 *vs* DY3**	Liver	Bray-Curtis	0.0925
	Muscle	Bray-Curtis	**0.03722**
	Gut microbiota	Weighted Unifrac	0.10095
**DY1&2 *vs* DY4**	Liver	Bray-Curtis	**0.00457**
	Muscle	Bray-Curtis	**0.02394**
	Gut microbiota	Weighted Unifrac	0.05735
**DY3 *vs* DY4**	Liver	Bray-Curtis	0.08869
	Muscle	Bray-Curtis	0.17267
	Gut microbiota	Weighted Unifrac	0.26656

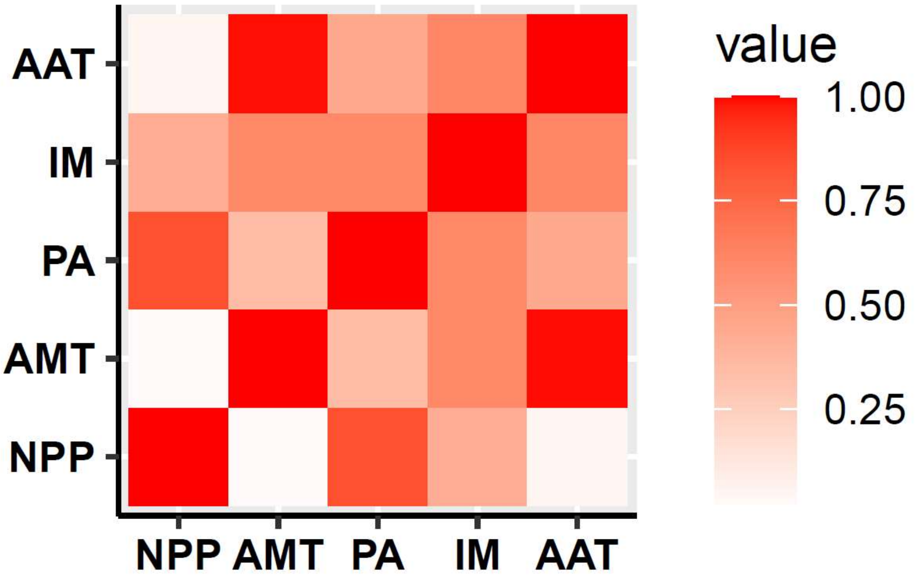

Figure S1 Correlations between climatic factors.

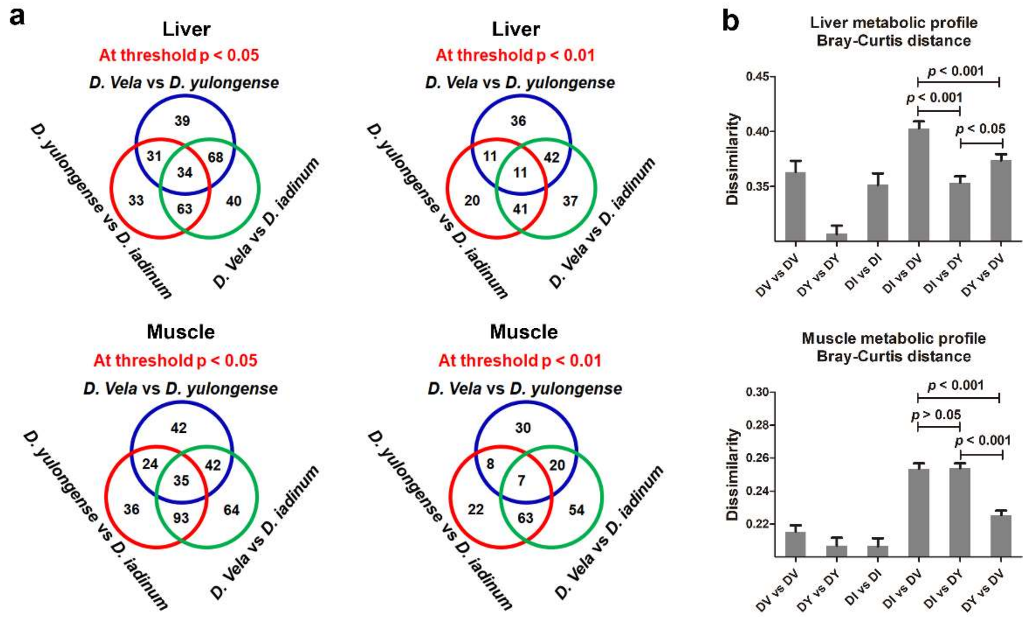

Figure S2 Interspecies comparison of organ metabolome. (a) Venn plots presenting the numbers of metabolites varied between species. (b) Bray-Curtis distances of metabolomes between species.

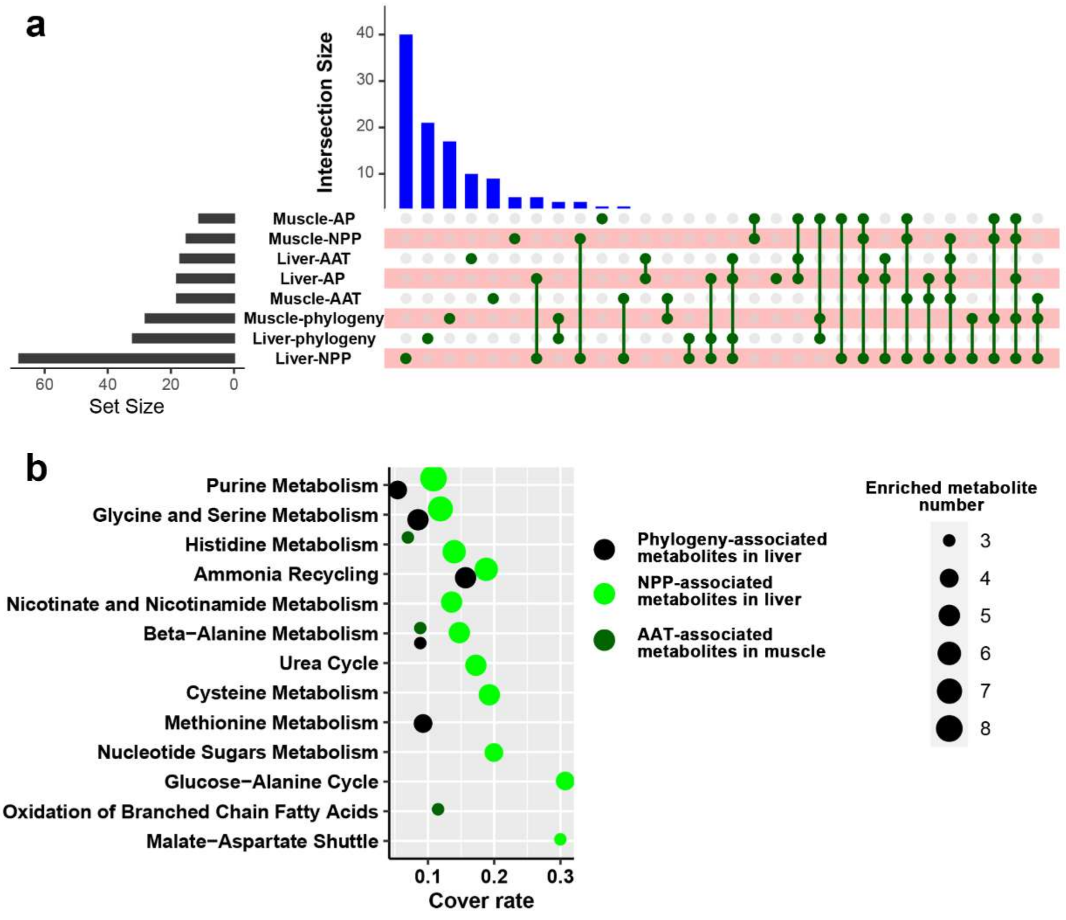

Figure S3 Metabolites varied with host phylogeny and climatic factors. (a) Upset plot presenting the numbers of metabolites associated with phylogeny and each climatic factor. (b) KEGG enrichement analysis based on metabolites associated with phylogeny and each climatic factor (MetaboAnalyst 5.0). The items meet $p < 0.01$ were displayed.

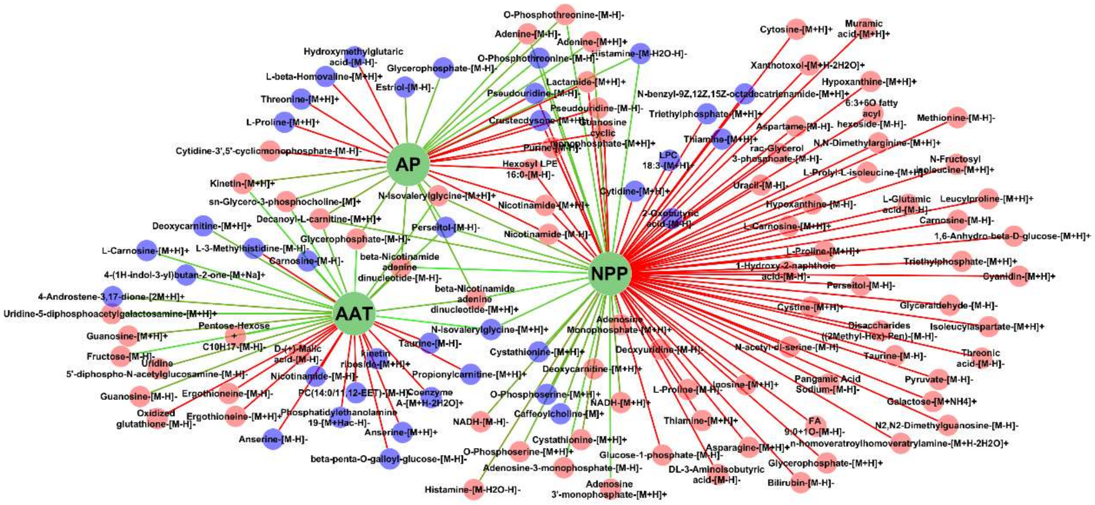

Figure S4 Network displaying the significant correlations ($q < 0.05$ for Pearson correlation and p < 0.05 for Spearman correlation) between metabolites and climatic factors. Red nodes: liver metabolites; blue nodes: muscle metabolites; green nodes: climatic factors; red edge, positive correlations; green edge, negative correlations.

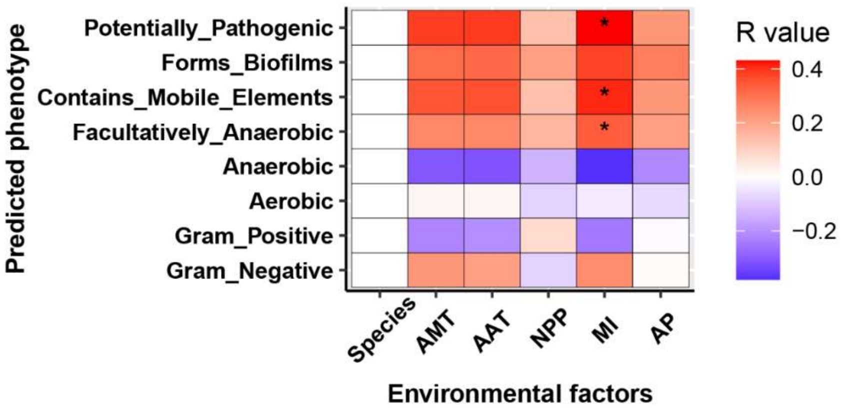

Figure S5 Heatmap presenting the associations between climatic factors and predicted bacterial phenotypes. *, $p < 0.05$ in ANOCOVA.

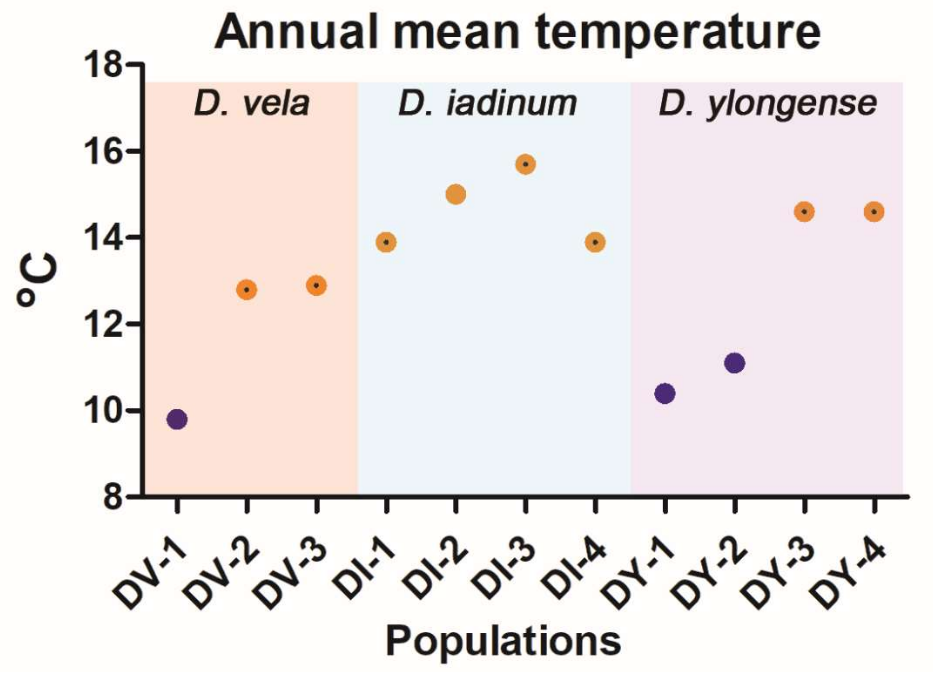

Figure S6 The annual mean temperatures of the collection sites for *D. vela* and *D. yulongense*.

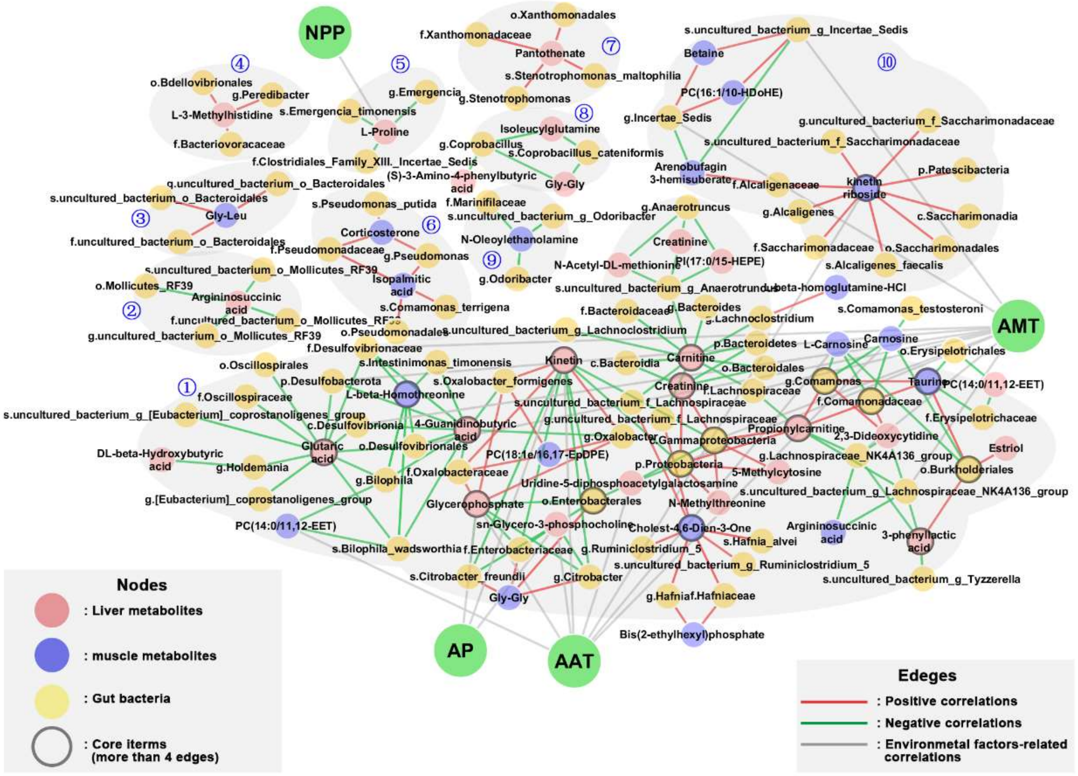

Figure S7 Networks displaying the significant correlations ($q < 0.05$ for Pearson correlation and $p < 0.05$ for Spearman correlation)

between metabolites, bacterial taxa, and climatic factors. Liver core metabolites (correlated with more than four bacterial taxa) included glycerophosphate, propionyl carnitine, kinetin, carnitine, creatinine, glutaric acid, 3-phenyllactic acid, and 4-guanidinobutyric acid. Muscle core metabolites included taurine, cholest-4,6-dien-3-one, kinetin-riboside, and beta-homothreonine. Core bacterial taxa were p.Proteobacteria, c.Gammaproteobacteria, o.Enterobacterales, f.Comamonadaceae, g.Comamonas, and o.Burkholderiales. Notably, metabolites associated with environmental AAT and bacteria associated with AMT were involved in the same correlation network.
